# Prediction of Autopsy Verified Neuropathological Change of Alzheimer’s Disease Using Machine Learning and MRI

**DOI:** 10.3389/fnagi.2018.00406

**Published:** 2018-12-10

**Authors:** Alexander Kautzky, Rene Seiger, Andreas Hahn, Peter Fischer, Wolfgang Krampla, Siegfried Kasper, Gabor G. Kovacs, Rupert Lanzenberger

**Affiliations:** ^1^Department of Psychiatry and Psychotherapy, Medical University of Vienna, Vienna, Austria; ^2^Department of Psychiatry, Danube Hospital, Medical Research Society Vienna D.C., Vienna, Austria; ^3^Department of Radiology, Danube Hospital, Vienna, Austria; ^4^Institute of Neurology, Medical University of Vienna, Vienna, Austria

**Keywords:** Alzheimer’s disease, machine learning, neuropathology, MRI, neuroimaging

## Abstract

**Background:** Alzheimer’s disease (AD) is the most common form of dementia. While neuropathological changes pathognomonic for AD have been defined, early detection of AD prior to cognitive impairment in the clinical setting is still lacking. Pioneer studies applying machine learning to magnetic-resonance imaging (MRI) data to predict mild cognitive impairment (MCI) or AD have yielded high accuracies, however, an algorithm predicting neuropathological change is still lacking. The objective of this study was to compute a prediction model supporting a more distinct diagnostic criterium for AD compared to clinical presentation, allowing identification of hallmark changes even before symptoms occur.

**Methods:** Autopsy verified neuropathological changes attributed to AD, as described by a combined score for Aβ-peptides, neurofibrillary tangles and neuritic plaques issued by the National Institute on Aging – Alzheimer’s Association (NIAA), the ABC score for AD, were predicted from structural MRI data with RandomForest (RF). MRI scans were performed at least 2 years prior to death. All subjects derive from the prospective Vienna *Trans-*Danube Aging (VITA) study that targeted all 1750 inhabitants of the age of 75 in the starting year of 2000 in two districts of Vienna and included irregular follow-ups until death, irrespective of clinical symptoms or diagnoses. For 68 subjects MRI as well as neuropathological data were available and 49 subjects (mean age at death: 82.8 ± 2.9, 29 female) with sufficient MRI data quality were enrolled for further statistical analysis using nested cross-validation (CV). The decoding data of the inner loop was used for variable selection and parameter optimization with a fivefold CV design, the new data of the outer loop was used for model validation with optimal settings in a fivefold CV design. The whole procedure was performed ten times and average accuracies with standard deviations were reported.

**Results:** The most informative ROIs included caudal and rostral anterior cingulate gyrus, entorhinal, fusiform and insular cortex and the subcortical ROIs anterior corpus callosum and the left vessel, a ROI comprising lacunar alterations in inferior putamen and pallidum. The resulting prediction models achieved an average accuracy for a three leveled NIAA AD score of 0.62 within the decoding sets and of 0.61 for validation sets. Higher accuracies of 0.77 for both sets, respectively, were achieved when predicting presence or absence of neuropathological change.

**Conclusion:** Computer-aided prediction of neuropathological change according to the categorical NIAA score in AD, that currently can only be assessed post-mortem, may facilitate a more distinct and definite categorization of AD dementia. Reliable detection of neuropathological hallmarks of AD would enable risk stratification at an earlier level than prediction of MCI or clinical AD symptoms and advance precision medicine in neuropsychiatry.

## Introduction

Alzheimer’s disease (AD) as the most common form of dementia is estimated to affect over 30 million people worldwide, making it one of the most burdensome diseases for individuals, their relatives as well as society (Takizawa et al., 2015). The clinical diagnosis of AD dementia requires the presence of manifest symptoms such as progressive cognitive impairment. Diagnosis based on cognitive testing and patients’ history or even based on symptoms described by relatives is common in clinical routine. However, lesions attributable to AD may antecede these clinical symptoms by years. Furthermore, certain neuropathological changes attributed as hallmark lesions of AD have been specifically associated to AD dementia, while clinical signs of dementia can be caused by several diseases (Albert et al., 2011; McKhann et al., 2011). Autopsy and neuropathologic examination are therefore considered the gold standard of AD diagnostics and allow for assessment of AD years to decades before clinical onset (Sperling et al., 2011). An overhauled staging system for these changes has recently by provided by the “National Institute on Aging – Alzheimer’s Association” (NIAA), assessing the decisive neuropathologic features of AD (Montine et al., 2012). This requires evaluation of extracellular deposits of β-amyloid peptides (Aβ) or senile plaques, neurofibrillary degeneration in the form of tangles (NFTs) containing hyperphosphorylated tau protein, and scoring of neuritic plaques, representing a subset of senile plaques surrounded by tau-containing dystrophic neurites (Braak and Braak, 1991; Tiraboschi et al., 2004). Intermediate or high neuropathological change has been shown to sufficiently explain clinical symptoms while low-grade changes might antecede symptoms substantially.

Considering rising life expectancies together with increasing prevalence rates of dementia, computer-aided methods for early and reliable prediction of AD dementia have been asked for to overcome the shortcomings of clinical diagnostics. Structural magnetic resonance imaging (MRI) has emerged as a promising tool for *in vivo*, non-invasive identification of AD-associated brain alterations that could mark patients at risk of progression to dementia early on. Acquisition of structural MRIs is rather simple, widely available and no explicit design is required. Multivariate pattern analysis and machine learning algorithms show decisive advantages over conventional, univariate statistics and allow classification of phenotypes based on MRI data. Exploiting these new statistical approaches capable of processing large amounts of data independently of predefined hypotheses, several reports of high accuracies ranging from 0.75 to 0.96 emerged especially in the field of MRI (Fayed et al., 2016; Ardekani et al., 2017; Beheshti et al., 2017; Long et al., 2017). Thereby, research was focused on early detection of subjects at high risk for clinical manifestation of AD dementia. These studies suggested sufficient predictive power to distinguish healthy elderly from mild cognitive impairment (MCI) or AD and predict conversion from MCI to fully established AD dementia. Thereby, voxel-based whole brain as well as region of interest (ROI) based approaches have been applied with a broad range of algorithms that usually depend on a training sample to allow prediction for a test dataset. Several algorithms have successfully been deployed, including RandomForest (RF) and Support Vector Machines (SVM) (Aguilar et al., 2013). The corresponding predictors have usually been based on feature intensity, tissue density or shape.

These recent results have advocated the potential of computer-aided diagnostics and precision medicine by allowing detection of patients at risk of developing AD dementia at a preclinical or an early clinical level. Nevertheless, only one prediction model for neuropathologically verified dementias has been proposed yet (Harper et al., 2016). Considering that neuropathological lesions are more distinct and definite than clinical symptoms, as the latter can be caused by a variety of alterations unrelated to AD, we conducted a machine learning investigation applying RF to predict NIAA AD scores from structural MRI data in a ROI based approach. The main goal was to establish a computer-aided tool to support clinical risk-assessment and diagnosis at an earlier and more distinct level than MRI-based prediction of clinical symptoms of AD dementia. These data were collected irrespectively of clinical symptoms in a sample deemed representative for elderly in Austria and may therefore allow precise stratification of subjects by neuropathological lesions, resulting in earlier detection of patients at risk for developing AD dementia.

## Materials and Methods

### Subjects

All 68 subjects enrolled in this study derive from the prospective Vienna *Trans-*Danube Aging (VITA) study that has been described previously (Fischer et al., 2002; Kovacs et al., 2013). The VITA study targeted all people born between May 1925 and June 1926 in the 21st and 22nd districts of Vienna, irrespective of clinical symptoms or diagnoses. Of 1750 registered inhabitants, 697 underwent baseline examination in the year 2000, being at 75–76 years of age. Subjects were subsequently invited to follow-ups. Cranial MRI measurements were conducted for all eligible subjects. Clinical investigations were performed, including blood sampling, neuropsychological testing and psychiatric examinations. Irrespective of neuropsychiatric impairment, all subjects participating in the study that died in the Danube Hospital of Vienna between 2001 and 2016 were brought to neuropathological examination. All 68 subjects of whom MRI as well as neuropathologic data were available have been allocated to this investigation and 49 showed sufficient MRI data quality for further statistical analysis.

All procedures have been approved by the local Ethics Committee of the Medical University of Vienna. For details on sex, age and AD neuropathological change, please also consider Table 1.

**Table 1:** Baseline characteristics including the age of death and therefore neuropathological examination, sex distribution as well as NIAA AD score.

Baseline characteristics (*n* = 49)	
NIAA AD score *No, low, and high* neuropathological change	16 / 15 / 18
Sex (female)	26
Age at death (±SD)	82.8 (±2.9)


*This sample does not differ significantly from the subjects excluded due to poor MRI quality (*n* = 19) regarding any of these variables. NIAA AD, National Institute on Aging – Alzheimer’s Association Alzheimer’s disease score; SD, standard deviation.*

### Neuropathologic Examination

Neuropathologic examination has been described in detail previously (Kovacs et al., 2013). To ensure data quality and rule out bias, all cases were examined by at least two certified neuropathologists using a multi-headed microscope. For evaluation of neuropathology, formalin fixed, paraffin-embedded tissue blocks of 2.5 × 2.0 cm were used. Samples of frontal, cingular, temporal, parietal, occipital cortex and white matter, anterior and posterior hippocampus, caudate nucleus, accumbens nucleus, putamen, globus pallidus, thalamus, mesencephalon, pons, medulla oblongata, cerebellar anterior vermis and cerebellar hemisphere and dentate nucleus were included in these blocks. Staining was performed with hematoxylin, eosin, Luxol fast blue and nuclear fast red as well as Bielschowsky and Gallyas. For immunohistochemistry, monoclonal antibodies, including phospho-Tau, phospho-TDP43, Aβ, α-synuclein, p62 and ubiquitin were applied; for a detailed description see also (Kovacs et al., 2013).

### Neuropathologic Variables

**FIGURE 1:**
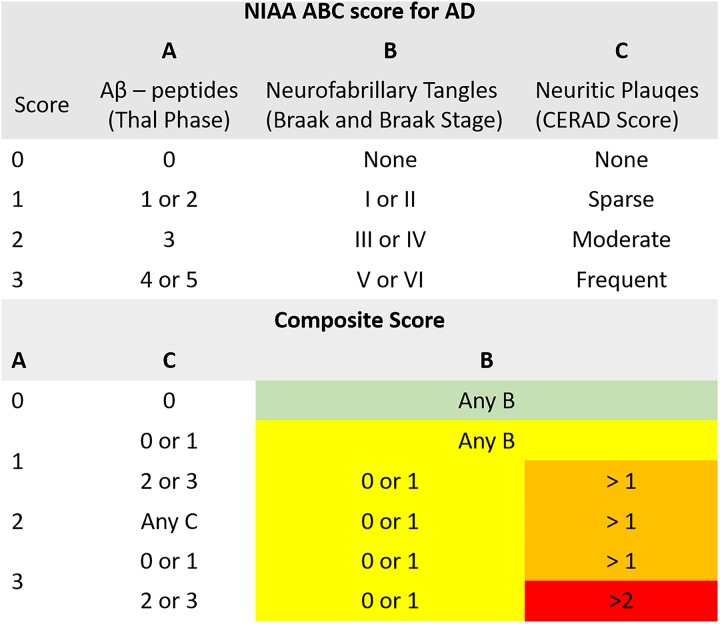
Illustration of the composite “National Institute on Aging – Alzheimer’s Association” (NIAA) ABC score for neuropathological change of Alzheimer’s disease (AD). The score comprises hallmark lesions attributed to AD, **(A)** Aβ-peptides according to Thal phase, **(B)** neurofibrillary tangles described by Braak and Braak stage and **(C)** neuritic plaques described by the CERAD score. While the ABC score issued by the NIAA has four levels, describing absence of (green color, *n* = 16) or presence of low (yellow color, *n* = 15), intermediate (orange color) or high (red color) neuropathological change, it was collapsed to three levels for this analysis to ensure sufficient group sizes. Thereby, intermediate and high change were merged into one group (*n* = 18).

For the neuropathologic assessment of AD, the NIAA guidelines were applied (Montine et al., 2012). Neuropathologic change was classified according to the “ABC coding system” of the NIAA, which comprises Aβ-peptides assessed with a modified version of Thal phases (Thal et al., 2002), NFTs assessed with a condensed version of the by Braak and Braak staging (Braak and Braak, 1991; Nagy et al., 1998; Braak et al., 2006), and neuritic plaques assessed according to the “Consortium to Establish a Registry for AD” (CERAD) protocol (Masliah et al., 1990, 1993). Three groups were determined based on detection of (1) no (*n* = 16), (2) low (*n* = 15), (3) intermediate or high (*n* = 18) neuropathologic changes. Merging of subjects with intermediate and high changes was necessary to ensure sufficient and comparable group sizes. Considering that intermediate and high changes have been implied to sufficiently explain clinical symptoms of cognitive impairment while low changes might precede these by years, the resulting three-leveled neuropathological outcome variable was practicable for the study goals (Hyman et al., 2012). For a schematic depiction of the NIAA score for AD and the three-leveled adaption predicted in this analysis please also refer to Figure 1.

### MRI

All subjects featured in this analysis underwent one MRI measurement at the age of 75–76 years. Considering the mean age of death (83 ± 3), on average MRI scans were performed 7–8 years prior to death. Thereby, a 1.0-Tesla unit (Siemens Impact Expert; Siemens Medical Systems, Inc., South Iselin, NJ) and a circular polarized skull coil were used. Coronary T1-weighted gradient echo MPRAGE sequence (matrix: 256 × 240, voxel size: 1 × 1 mm, slice thickness: 1 mm, 200 slices), T2-weighted Turbo Spin Echo, transverse proton density as well as thin-section inversion recovery sequence in the olfactory region were obtained for all subjects.

Nineteen patients had to be excluded from the subsequent analyses as poor MRI data quality due to motion, poor contrast or poor coverage prohibited reliable application of FreeSurfer software. Hence, 49 patients could be included for the statistical analyses.

### Data Preprocessing: Surface- and Volume Based Analysis

The standard procedure for the FreeSurfer software suite^1^ was used for cortical and subcortical assessment, as described previously (Seiger et al., 2016). Recent work indicated excellent performance of automated software like FreeSurfer for detection of structural alterations in AD, making it secondary only to post-mortem assessment (Seiger et al., 2018). In short, every volume of a subject was registered to the Talairach atlas via affine registration in the cortical based pipeline (Dale et al., 1999; Fischl et al., 1999). Applying a deformable template model, skull stripping was performed subsequent to bias field correction (Segonne et al., 2004). Hemisphere separation as well as cerebellum and brain stem removal were integrated in that step. After white matter segmentation, white and pial surfaces were estimated. For calculation of thickness of each cortical location, the distance between these surfaces was computed (Fischl and Dale, 2000). While Talairach registration and bias field correction were shared, different algorithms were used for labeling of subcortical tissue classes, as published previously (Fischl et al., 2002, 2004). To ensure high quality of segmentations, all the cortical and subcortical volumes were visually inspected after the automated streams. The data was subsequently partitioned to 134 ROIs according to the Desikan–Killiany atlas and the default segmentation implemented in FreeSurfer. These consisted of 66 subcortical as well as 68 cortical ROIs (34 for each hemisphere) (Desikan et al., 2006).

### Statistics

The NIAA score for AD was predicted from structural MRI data, also considering age of death and sex as predictors. Primary analyses were performed using the machine learning algorithm “RF” as provided by the synonymous package for the statistical software “R” (Liaw and Wiener, 2002). As there is no gold standard which classification algorithm may be most useful for the dataset at hand and RF is known for a risk to produce over-optimistic results, we also computed a SVM model for prediction of NIAA AD score.

**FIGURE 2:**
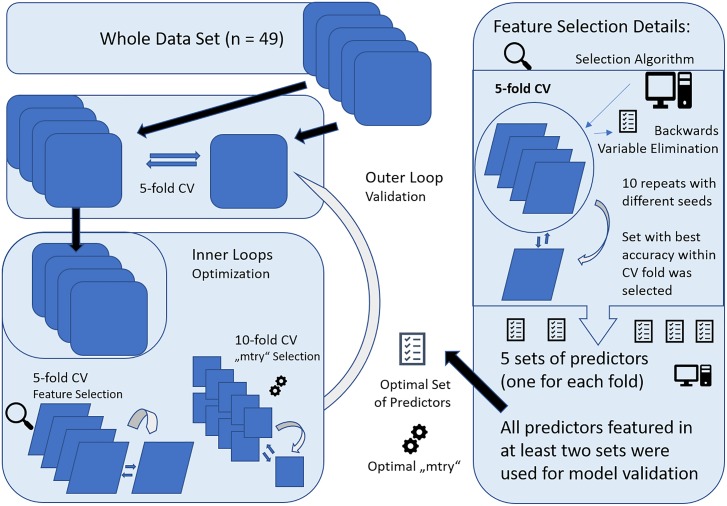
Graphical representation of the nested cross-validation (CV) design, consisting of a inner and outer loop. The inner loop was performed for model optimization with feature selection and setting of optimal parameters (“mtry”) for RandomForest (RF). For feature selection, fivefold CV was applied and all variables selected more than once across the runs were included within 10-fold CV for “mtry” selection. The resulting model was then applied to the validation set, forming the outer loop. This was repeated for all folds of the fivefold CV design for outer loop. Finally, the whole nested CV procedure was repeated 10 times and results were averaged.

Prediction was performed with nested cross-validation (CV) design that is illustrated in Figure 2. Nested CV is regarded as the gold-standard method if no independent dataset for validation is available and prevents circular analysis and other information leaks from the training to the validation models (Varoquaux et al., 2017). Thereby, data are first split into n-1 training sets and a test set in a n-fold CV design. Here, fivefold CV was applied to ensure sufficiently large test sets of 9–10 subjects. The training data or decoding set, consistent of 80% of the full data set, was used for model optimization, including hyperparameter tuning and feature selection applying further CV within the decoding set, also referred to as inner loops of the nested CV. After optimal parameters were set, the decoding set with optimal parameters was used for prediction in the test set, forming the outer loop of the nested CV design. This procedure is repeated for every fold of the outer loop, resulting in five different models with specific sets of predictors and hyperparameters determined in the respective inner loops. Accuracy is reported according to the predictive outcome of these five models. To account for variability of these models, the whole nested CV procedure was repeated ten times and average accuracy with SD is reported.

Within the inner loop, the algorithm “vaeSelRF” was used for feature selection as implemented in the eponymous package for “R.” “varSelRF” performs backward variable elimination aimed at detecting small sets of non-redundant variables to allow optimal prediction performance. The most informative features are selected by minimizing the out-of-bag prediction error by subsequently deleting the least important of the 134 predictors. Feature selection was performed in a fivefold CV design, with 10 runs with random starting seeds for each fold of the CV. The set of predictors with optimal accuracy in the test data was then retrieved and all features that were comprised in at least two of the five optimal sets from all fivefolds were used for validation. Applying this design, only predictors that show promising results for generalizability and some constancy get selected.

Concerning the details for RF variable importance measurement, the variables contributing most to accuracy of prediction of the NIAA AD score show the highest importance values measured by mean decrease in Gini index (MDG). RF computes regression trees by applying rearranged values for each variable in out-of-bag samples. The null hypothesis (H0) is challenged whenever a rearranged predictor variable decreases Gini values. Consequently, decrease in Gini therefore displays the contribution of each predictor to the homogeneity of branches and nodes of classification trees and values range from 0 for complete homogeneity to 1 for heterogeneity. Finally, summed and normalized changes for all nodes split up by a specific predictor are expressed in MDG values. As an increase in MDG signifies higher purity of the resulting nodes compared to original nodes, high MDG is an indicator for the importance of a specific feature for the prediction accuracy.

Concerning model parameters, the number of trees to grow (“ntree”) was set at 3000 to enable multiple predictions for all observations. While RF does not require tuning of hyperparameters, optimizing the number of features available for each split (“mtry”) can significantly increase model performance. A higher number of features allowed at each node leads to higher flexibility of the tree but also increases diversity of 3000 individual trees. Finding the optimal balance is data-dependent and requires tuning. For variable selection, the general standard of applying the square root of the number of predictors was used for “mtry.” After determination of the most informative features, 10-fold CV was performed in the decoding sets to find optimal “mtry” values for model validation with the “caret” package for “R.”

Finally, the optimal “mtry” and all variables selected by the inner loops were applied for training in the decoding sample and tested on the validation sample.

Concerning the alternative prediction model with SVM, the same nested CV design was applied. After feature selection with RF as described above, hyperparameters c and sigma were tuned with the “caret” package for “R” (c ranging from 0.01 to 100, sigma ranging from 0.01 to 0.9), similar in concept to “mtry” tuning for RF.

There is no established method of power calculation for RF. Research indicated stable predictive capabilities of RF and comparable machine learning algorithms when enough observations and no missing data are accounted for, regardless of the number of variables surpassing that of observations (Chen et al., 2011; Roetker et al., 2013). Therefore, RF can be expected to handle a ratio of 49 observations to 134 predictors.

To compare the results produced by RF to conventional multivariate statistics, the ten top scoring predictors of the variable selection algorithm were also analyzed with a mixed model as included in the “lmne” package of “R.” Subject served as the random factor and NIAA AD score, ROI and their interaction were included as fixed factors. Results were Bonferroni corrected (for number main and interaction effects). To identify the significant ROI, *post hoc* ANOVA was performed for each of the ten ROI with an uncorrected *p*-value threshold of 0.05.

The datasets analyzed in this study are available from the corresponding author on reasonable request.

## Results

### Variable Selection

The feature selection algorithm mostly suggested five variables as the most informative number of predictors (60% of optimal feature sets), with a maximum of 49 suggested variables. The average error for out-of-bag prediction with the optimal set of predictors was at 0.33 (±0.06). A high agreement of features selected at least twice was observed within the CV models of the inner loops. Over the whole nested CV runs a higher number of features selected at least twice was observed, ranging from 24 to 29 variables over the ten repeats.

**FIGURE 3:**
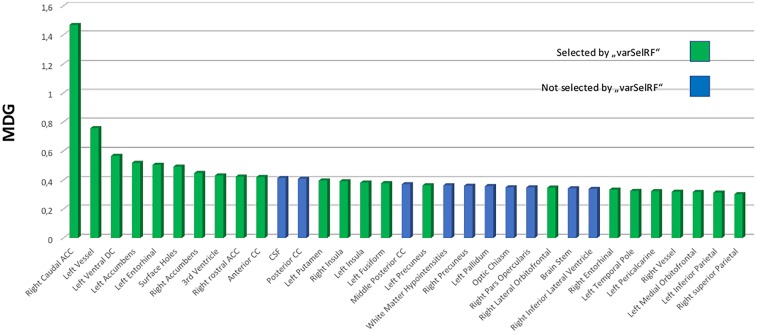
Variable importance measurement from RF by mean decrease in Gini (MDG) for the total data set (*n* = 49). The 25% top scoring out of all 134 predictors are portrayed and ordered by declining contribution to prediction quality to the RF model. Green coloring indicates that these predictors were selected among the most effective predictors for the “National Institute on Aging-Alzheimer’s Association” score for neuropathological change by backward variable selection performed with the “varSelRF” algorithm for the statistical software “R.” RF, RandomForest; MDG, mean decrease in Gini index; CC, corpus callosum; DC, diencephalon.

Ten ROI were consistently selected and therefore were of highest informative value among the 134 ROIs included. The caudal anterior cingulate gyrus was always comprised in the optimal feature sets. Furthermore, there was a high agreement for the right rostral anterior cingulate and inferior parietal gyrus, left entorhinal cortex, left nucelus accumbens, anterior corpus callosum, left ventral diencephalon (DC), left vessel, left precuneus as well as the “FreeSurfer” parameter “surface holes” as the most valuable features for prediction of the NIAA AD score. For comparison to the automated variable selection provided by “VarSelRF,” the most influential predictors according to a random call of the conventional importance function of RF were also plotted for the whole data set, as presented in Figure 3. The 10 ROIs most frequently suggested by the automated function were all featured within the top 15 predictors of the conventional importance measurement and 79% of the predictors repeatedly suggested by the feature selection algorithms scored in the upper quartile. Age at death and sex scored low in importance measurement but were included in all models.

### Mixed Model and MRI Results

Neither total gray matter, nor estimated total intracranial volume differed significantly between groups according to NIAA AD score (*p* > 0.05). The mixed model analysis revealed a significant interaction effect of NIAA AD score and ROI (corrected *p* = 0.039, *F* = 2.37) in addition to the expected main effect of ROI. The *post hoc* ANOVA analyses produced significant associations for the entorhinal thickness of the left hemisphere (*p* = 0.040, *F* = 4.451), the caudal anterior cingulate of the right hemisphere (*p* = 0.042, *F* = 4.383), the anterior corpus callosum (*p* = 0.016, *F* = 6.311) and the left ventral diencephalon (*p* = 0.027, *F* = 5.239), all of which were also selected among the most discriminative variables by the feature selection algorithm. Results of the conventional statistics are also reported in Table 2.

**Table 2:** Mixed model results for the ten highest scoring ROI for classification of NIAA AD score and *post hoc* ANOVA results for all associated ROI.

	DF	DF
(A) Predictor	“*numerator*”	“*denominator*”	*F*-value	*p*-value
NIAA AD score	1	47	1.590	n.s.
ROI	9	423	1175.085	<0.0001
ROI ^∗^ NIAA AD score	32	1408	2.372	0.039

**(B) *Post hoc* analyses**			***F***-**value**	***p***-**value**

Anterior corpus callosum			6.311	0.016
Left ventral diencephalon			5.239	0.027
Entorhinal thickness LH			4.451	0.040
Caudal anterior cingulate thickness RH			4.383	0.042


*Interaction effects are marked with^∗^, mixed model results were corrected for multiple testing. ROI, Region of interest; AD, Alzheimer’s disease; NIAA, National Institute on Aging – Alzheimer’s Association; DF, Degrees of Freedom; LH, Left Hemisphere; RH, Right Hemisphere.*

**FIGURE 4:**
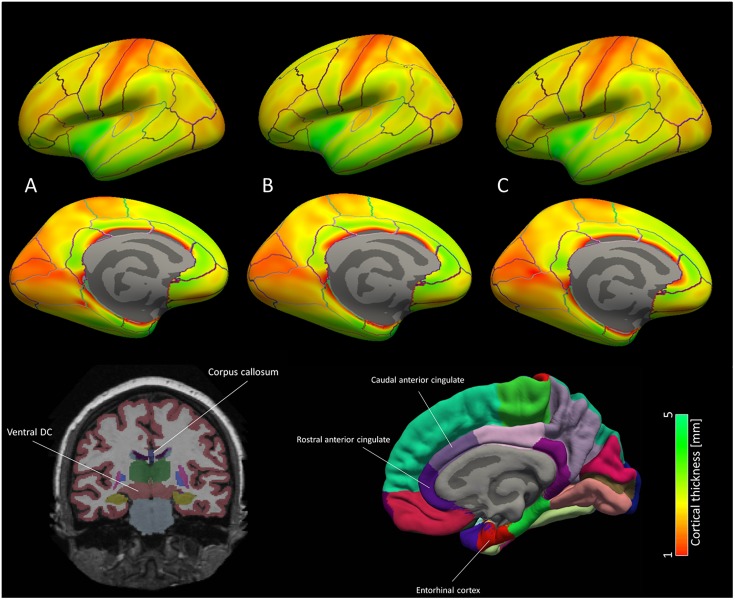
Brain map for the cortical ROI used for the machine learning classification models. Average cortical thickness values for the three groups according to NIAA AD score [**(A)** no change, **(B)** low change and **(C)** intermediate to high change] are portrayed for each hemisphere. According to machine learning and mixed model results, discriminative patterns for the NIAA AD score may be driven primarily by thickness of the entorhinal cortex and caudal anterior cingulate cortex as well subcortical ROI left ventral diencephalon and anterior corpus callosum, which are depicted schematically below the brain map. In the brain map, only cortical ROI are shown.

All these ROI showed a decline in mean cortical thickness with increase of NIAA AD score, for details see also Table 3. For a brain map showing average cortical thickness for all ROI for each of the three groups according to NIAA AD score, please refer to Figure 4.

**Table 3:** Average critical thickness for all three groups according to the NIAA ABC score for neuropathological change of AD for all significantly associated ROI of the *post hoc* analyses as well as for total intracranial and gray matter volume.

	NIAA AD score		
		
ROI	No	Low	Intermediate and high
Anterior corpus callosum	735.83	703.59	604.74
Left ventral diencephalon	3607.32	3451.11	3295.52
Entorhinal thickness LH	3.04	2.97	2.79
Caudal anterior cingulate thickness RH	2.44	2.35	2.25
Estimated total intracranial volume	1509229.23	1547219.74	1502761.30
Total gray matter volume	490399.48	504419.15	493171.61


*Groups did not differ significantly in overall gray matter or intracranial volume. ROI, Region of interest; AD, Alzheimer’s disease; NIAA, National Institute on Aging – Alzheimer’s Association; DF, Degrees of Freedom; LH, Left Hemisphere; RH, Right Hemisphere.*

### Prediction Results

For the 10-fold CV models with optimal feature sets (inner loop, “mtry optimization”), an average accuracy of 0.62 (±0.05) could be achieved for prediction of the NIAA score. Optimal “mtry” ranged from 2 to 19 across models. The sensitivity for predicting any neuropathological change was at 0.88, while the specificity was at 0.5. The resulting accuracy for detection of any neuropathological change was at 0.77 (±0.03).

For model validation within the outer loop, the average accuracy for classification of the NIAA score was at 0.61 (±0.02) and 0.77 (±0.04) for detection of any neuropathological change. For binomial evaluation, the positive predictive value (PPV) was at 0.79, indicating the probability of correct prediction of present neuropathological change. The negative predictive value (NPV) was at 0.73.

Confusion matrices for the categorical models are displayed in Table 4. For a detailed overview of binary prediction outcome with all evaluation parameters for each model, please see Table 5.

**Table 4:** Categorical evaluation of RandomForest (RF) prediction models.

Confusion matrices for categorical evaluation							
*NIAA-AD score* (three levels)							

Decoding sets *(n = 39)*				Validation sets *(n = 49)*			
	
Predicted				Predicted			
	
Count	No	Low	High	Count	No	Low	High
No	8	8	1	No	11	7	1
Low	1	4	5	Low	2	4	4
High	0	0	12	High	0	0	15
	Acc. = 0.62 ± 0.05				Acc. = 0.61 ± 0.02		


*NIAA AD score equivalent to “no,” “low,” and “intermediate to high” change was used for classification and confusion matrices are provided. Comparable accuracies were computed for the prediction in the decoding and the validation samples. The nested cross-validation (CV) procedure was repeated 10 times and average accuracies with standard deviations are reported. For the matrices, exemplary results with comparable accuracy are shown. NIAA AD, National Institute on Aging – Alzheimer’s Association Alzheimer’s disease score.*

**Table 5:** Binary evaluation of RF prediction models.

Binomial Evaluation *Change vs. No Change*	Sensitivity	Specificity	PPV	NPV	Accuracy
**Decoding Sample** *(n = 39) 10-fold CV of the inner loop*					
*averaged over 10 repeats*	0.88	0.5	0.8	0.66	0.769 (±0.03)
**Decoding Sample** *(n = 39)* **+ Validation Sample** *(n = 10) fivefold CV of the outer loop*					
*averaged over 10 repeats*	0.91	0.5	0.79	0.73	0.774 (±0.04)


*Neuropathological change was compared to no change and performance evaluators sensitivity, specificity, PPV, NPV, and accuracy are provided for the models of the inner (decoding samples) and outer (validation samples) loop of the nested CV. Overall, comparable accuracies around 0.77 were reached across the models. All results are averaged over 10 repeats of the whole nested CV procedure. PPV, positive predictive value; NPV, negative predictive value; CV, cross-validation; RF, RandomForest.*

The alternative prediction model computed with SVM produced a lower accuracy of 0.51 (±0.04) for three-leveled NIAA AD score and an accuracy of 0.74 (±0.05), almost equivalent to the RF model, for prediction of absence or presence of neuropathological change.

## Discussion

Various machine learning and multivariate data analysis methods have been introduced to AD research within the last decade. Usually they aimed at automated prediction of clinical phenotypes based on disease-related data patterns. Mostly, the goal has been discrimination of AD dementia patients or MCI from healthy elderly and prospective prediction of patients who show progression from MCI to AD dementia (Falahati et al., 2014; Salvatore et al., 2016). While promising results for prediction of clinical outcomes have been reported before, no algorithm was established for classification of MRI data for prediction of neuropathological AD scales. Exploiting machine learning algorithms RF and SVM, we were able to generate a model for successful prediction of AD neuropathological change in 77% of cases.

Concerning separation of healthy elderly from AD dementia patients, the strongest results have been obtained. Overall decreased brain volumes in AD dementia patients compared to healthy controls enabled consistently accuracies above 0.90 (Kloppel et al., 2008; Casanova et al., 2011; Willette et al., 2014; Zhou et al., 2014; Beheshti et al., 2016, 2017). While multivariate or whole-brain approaches yielded best results, especially alterations in the hippocampus, amygdala, cingulate and entorhinal cortex as well as thalamus, putamen and pallidum have been associated with AD (Scahill et al., 2002; Fox and Schott, 2004; Cho et al., 2014). Differences between MCI and healthy controls on the other hand are less pronounced. Literature on detection of MCI among healthy elderly supports accuracies ranging from 0.71 to 0.91 and the hippocampus as well as the amygdala were highlighted as distinctive ROIs (Fan et al., 2008; Chupin et al., 2009). As some patients with MCI can be considered stable and do not show a progression to clinically manifest AD within a relevant timeframe (usually 18–36 month), models for distinguishing between stable and progressive MCI have been developed. Due to the more delicate differences between these groups, lower accuracies between 0.67 and 0.88 could be obtained (Fan et al., 2008; Querbes et al., 2009; Lillemark et al., 2014). Again, the hippocampus was especially predictive, and models based on several cortical and subcortical ROIs labeled via FreeSurfer showed advanced predictive capacity (Westman et al., 2012; Aguilar et al., 2013). Among these models, a whole-brain gray matter ROI deformation-based algorithm by Long et al. (2017) showed the best prediction outcome.

Contrary to previous investigations, the focus of this study was prospective detection of neuropathological change attributed to AD. Autopsy is still regarded as the gold-standard and only definite diagnostic tool for AD and hallmark lesions observable by neuropathologic examinations have been demonstrated to precede any clinical symptoms as cognitive impairment by years (Sperling et al., 2011; Montine et al., 2012, 2016). Furthermore, considering that most forms of dementia show mixed features and are etiologically less distinct than commonly expected, only neuropathological examination can rule out erroneous attribution of clinical symptoms to AD (Kovacs et al., 2013). These flaws of approaches implementing only clinical diagnosis as outcome variable for machine learning analyses has recently been criticized by a review on this topic (Salvatore et al., 2016). In fact, only one study applied machine learning to autopsy data, however, focused on speech changes in AD rather than early detection (Rentoumi et al., 2014). On the other hand, clinical symptoms divergent from neuropathology have been reported and not all patients with neuropathological change develop clinical symptoms. Consequently, an MRI-imaging based prediction model can at best assist clinical risk-assessment and diagnosis. Keeping this limitation in mind, an MRI-based prediction tool for neuropathological change may also be applicable to guide histopathological analysis.

Based on the successful implementation of machine learning algorithms in AD neuroimaging research, we expected a high accuracy for our prediction model. However, we predicted three-leveled categorical instead of the common binomial outcome, which is more penalizing as a chance level of 0.33% instead of the common 50% can be assumed. The rationale behind this was that distinguishing between low and intermediate or high neuropathological changes allows separation of potential clinical phenotypes as only intermediate to high changes can explain cognitive impairment in patients (Hyman et al., 2012; Montine et al., 2012). The observed accuracies of approximately 0.6 are substantially lower than those reported for clinical phenotypes, however, comparing just absence to presence of neuropathological changes increased the accuracy to 0.77. While these accuracies are still low for clinical application and lower than those for MCI or manifest AD, classification of neuropathological change allows even earlier and more definite risk stratification. Considering that curative or effectively arrestive treatment for AD is still lacking, detection of AD neuropathologic change years before they could become clinically relevant may facilitate effective prevention measures, e.g., by risk factor management (Hsu and Marshall, 2017).

Regarding the predictive features for this model, ten ROIs were selected consistently by variable importance measures and backward variable elimination. Most of the structures labeled by these ROIs have previously been associated with AD. The entorhinal and rostral as well as caudal anterior cingulate thickness ranked among the highest predictors and have consistently been associated with AD (Falahati et al., 2014; Salvatore et al., 2016). Both regions might be early markers for AD and interestingly, the entorhinal thickness measured pre-mortem by MRI has recently been associated with neurofibrillary tangles in neuropathologically assessed post-mortem AD brains (Thaker et al., 2017). The nucleus accumbens has also been suggested to show decreased activation and structural lesions in AD (Kazemifar et al., 2017; Lee et al., 2017). The FreeSurfer ROI left vessel and left VDC do not label a specific region but rather a conglomerate of structures that cannot be easily distinguished by MRI (Fischl, 2012). Left vessel describes lacunar alterations in putamen and pallidum, while the VDC is mostly comprising the hypothalamus, basal ganglia with subthalamic nuclei as well as geniculate nuclei, substantia nigra, red nucleus and mammillary body. Alterations of the basal ganglia have repeatedly been described for AD, however, these structures did usually not show high information criteria for machine learning (Serrano-Pozo et al., 2011; Risacher and Saykin, 2013; Van Dam et al., 2016). White matter alterations as suggested by selection of the anterior corpus callosum for classification, have been linked to AD by some studies (Bejanin et al., 2017; Lao et al., 2017). Reduced thickness of the anterior corpus callosum was also reported by a study assessing post-mortem brains affected by AD (Tomimoto et al., 2004). Finally, “surface holes” is not an anatomical ROI but a quality control parameter of FreeSurfer indicating the number of holes in the surface that were corrected by the algorithm for each subject. The selection of this marker was surprising and may be a false positive finding owed to the rather small data set. The average number of surface holes was about 20% higher in the groups with neuropathological changes (161 vs. 191, respectively), implying that subjects with AD hallmark lesions may be more difficult to process for automated algorithms as FreeSurfer. Therefore, surface holes may be a proxy marker for neuropathological chance.

The study sample with 49 subjects is high for a neuroimaging/neuropathology hybrid analysis, however, is low for machine learning application. In order to warrant stable performance and keep false positive findings low, we used a nested CV design with feature selection and parameter tuning in decoding sets. The confidence in our results is increased by comparable prediction performance across the ten repeats of nested CV. Furthermore, the overall similar outcomes for prediction of presence or absence of neuropathological change with SVM and RF models warrants some independency of our results from the machine learning algorithm applied. Nevertheless, validation in a bigger and independent sample is mandatory to prove the value of this model outside of our data. Another important limitation is the low quality of the MRI data used for this analysis as these were collected between 2000 and 2001 with a now outdated 1 Tesla scanner. As advantages of newer 3 or even 7 Tesla scanners are obvious, it is clear that the classification model would profit from high resolution imaging. On the other hand, FreeSurfer partly antagonizes the higher resolutions provided by higher Tesla scanners by down sampling all data to 1 mm. Furthermore, a comparative analysis did not show resounding advantages of higher resolution scanners (Seiger et al., 2015).

In synopsis, we successfully established a classification model for early prediction of post-mortem neuropathological change attributed to AD. Thereby, we addressed a clear shortcoming of previous research that solely based their predictions on clinical diagnosis of MCI or AD. Reaching an accuracy of 0.77 in our validation sample, the performance of this model is not fit for clinical application but represents a decisive step toward precision medicine in AD.

## Author Contributions

AK and RS were prepared the manuscript and responsible for data preparation and statistics. AH supervised all data related procedures and assisting in first level data preparation. PF and WK were involved in planning the study rationale and management of clinical and MRI data in the Danube Hospital, Vienna. SK advised on preparation the manuscript and was involved in planning the study design. GK was responsible for all neuropathological assessments. RL was responsible for supervision of all study related procedures and planning the study design.

## Conflict of Interest Statement

SK received grants/research support, consulting fees, and/or honoraria within the last 3 years from Angelini, AOP Orphan Pharmaceuticals AG, AstraZeneca, Eli Lilly, Janssen, KRKA-Pharma, Lundbeck, Neuraxpharm, Pfizer, Pierre Fabre, Schwabe and Servier. RL received travel grants and/or conference speaker honoraria from AstraZeneca, Lundbeck A/S, Dr. Willmar Schwabe GmbH, AOP Orphan Pharmaceuticals AG, Janssen-Cilag Pharma GmbH, and Roche Austria GmbH. The remaining authors declare that the research was conducted in the absence of any commercial or financial relationships that could be construed as a potential conflict of interest.

## Footnotes

1 http://surfer.nmr.mgh.harvard.edu/, version 5.3.0
